# Congenital lobar emphysema: a case report

**DOI:** 10.1186/1757-1626-2-67

**Published:** 2009-01-20

**Authors:** Asok Kumar Datta, Syamali Mandal, Jadab Kumar Jana

**Affiliations:** 1 Department of Pediatric Medicine, Burdwan Medical College and Hospital, Burdwan, West Bengal, India; 2 Department of Gynecology and Obstetrics, Burdwan Medical College and Hospital, Burdwan, West Bengal, India

## Abstract

Congenital lobar emphysema is a rare variety of congenital malformation of lung characterized by over distension of a lobe of a lung due to partial obstruction of the bronchus. We are reporting a neonate admitted in the pediatric emergency ward with the respiratory distress since 16^th ^day of life.

Investigation revealed the overexpansion of the left upper lobe with mediastinal herniation, shifting of the mediastinum to the opposite side and collapse of the ipsilateral lower lobe. The baby was treated with conservative treatment and the condition of the baby was improved.

## Background

Congenital lobar emphysema (CLE) is a rare malformation of lung development which may be the cause of respiratory insufficiency of the suckling child. It is caused by the hyper inflation of the lung lobe with compression of the normal lung parenchyma and contra lateral displacement of the mediastinum [1].

Over distension of the pulmonary lobe is secondary to partial bronchial obstruction [2]. Most common affected lobe is left upper lobe followed by right upper lobe and right middle lobe but any lobe may be affected. There is inspiratory air entry but collapse of the narrow bronchial lumen during expiration. The bronchial defect results in lobar air trapping.

Congenital lobar emphysema is an uncommon but potentially life threatening abnormality affecting infants. Patients often present within the first 6 months of life with recurrent respiratory distress [3]. Chest X-ray and CT scan of thorax are diagnostic and show the hyperluscent affected lobe with herniation of the lobe to the opposite side, shifting of the mediastinum to the opposite side and collapse of the remaining part of the ipsilateral lung [4].

Concomitant congenital heart disease (CHD) is not uncommon in CLE. In the literature a 12 to 20% concomitant rate is given. The presence of CHD, specially in infants with unusual respiratory distress symptom, should be kept in mind [5].

The management of congenital lobar emphysema has traditionally been surgical. Because of increased use of imaging, this lesion is frequently found in asymptomatic and mildly symptomatic children, prompting us to adopt a more conservative approach to these children [6].

## Case presentation

A 22^nd ^day old male neonate from a village Raina of the Burdwan district was referred to pediatrics emergency ward for respiratory difficulty for last1 week. There was no history of cyanosis, jaundice, convulsion and sign of heart failure.

He was from a poor socioeconomic status family,

Antenatal history of mother was uneventful, Ultrasonography was not done at that time. Baby was normally delivered in a rural hospital. Immediate postnatal period was normal.

Baby was breast fed and immunized at par.

On examination the baby was found in mild respiratory difficulty, respiration rate was 80 per minute, heart rate 130 per minute, facies normal, weight was 3.0 kg, length 55 cm and head circumference 35 cm. There was no cyanosis, jaundice or any signs of heart failure.

Examination of respiratory system revealed less movement of left upper chest, trachea was shifted to the right, vocal resonance was decreased in left side and on auscultation diminished breath sound was found on left side.

Other systems appeared normal.

**Figure 1:**
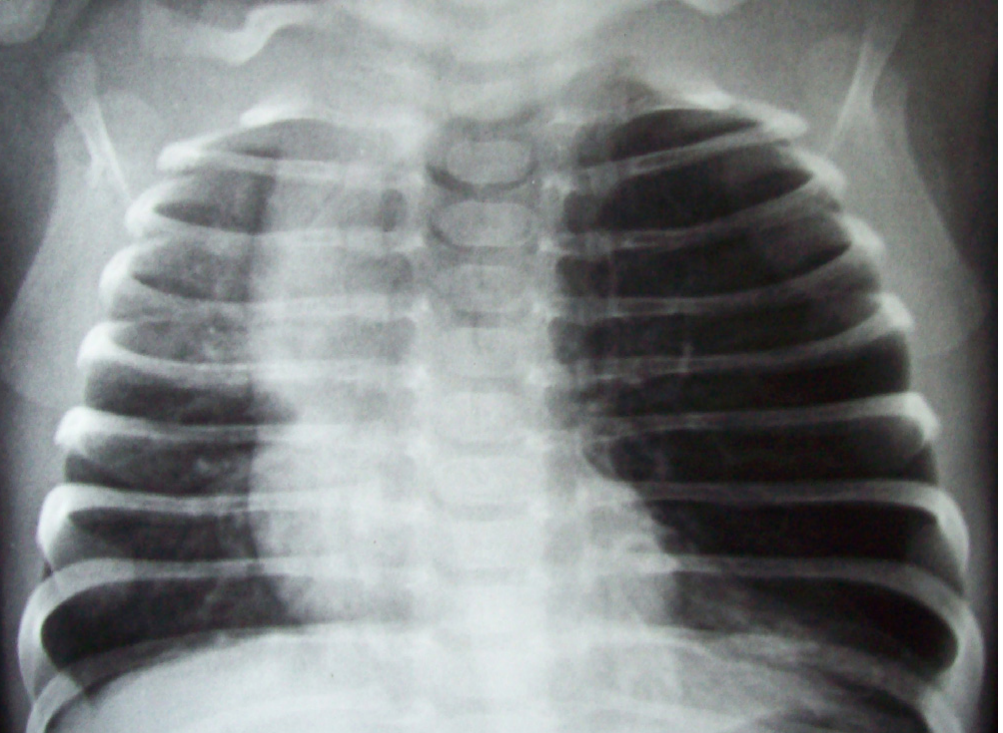
**Chest X-ray PA view**. Chest X-ray showed marked overdistension of the the left upper lobe with mediastinal shift to the right and collapse of the ipsilateral remaining lung field.

**Figure 2:**
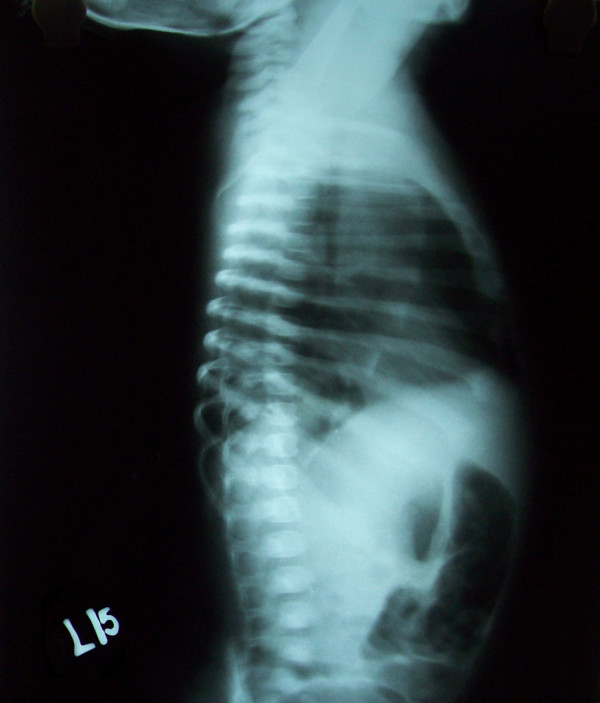
**Left lateral view of Chest X-ray**. On lateral view, the heart is displaced posteriorly with retrosternal luscency representing an anteriorly herniated lobe.

Chest X-ray showed marked overdistension of the the left upper lobe with mediastinal shift to the right and collapse of the ipsilateral remaining lung field (Figure 1). On lateral view,

The heart was displaced posteriorly with retrosternal luscency representing an anteriorly herniated lobe(Figure 2).

**Figure 3:**
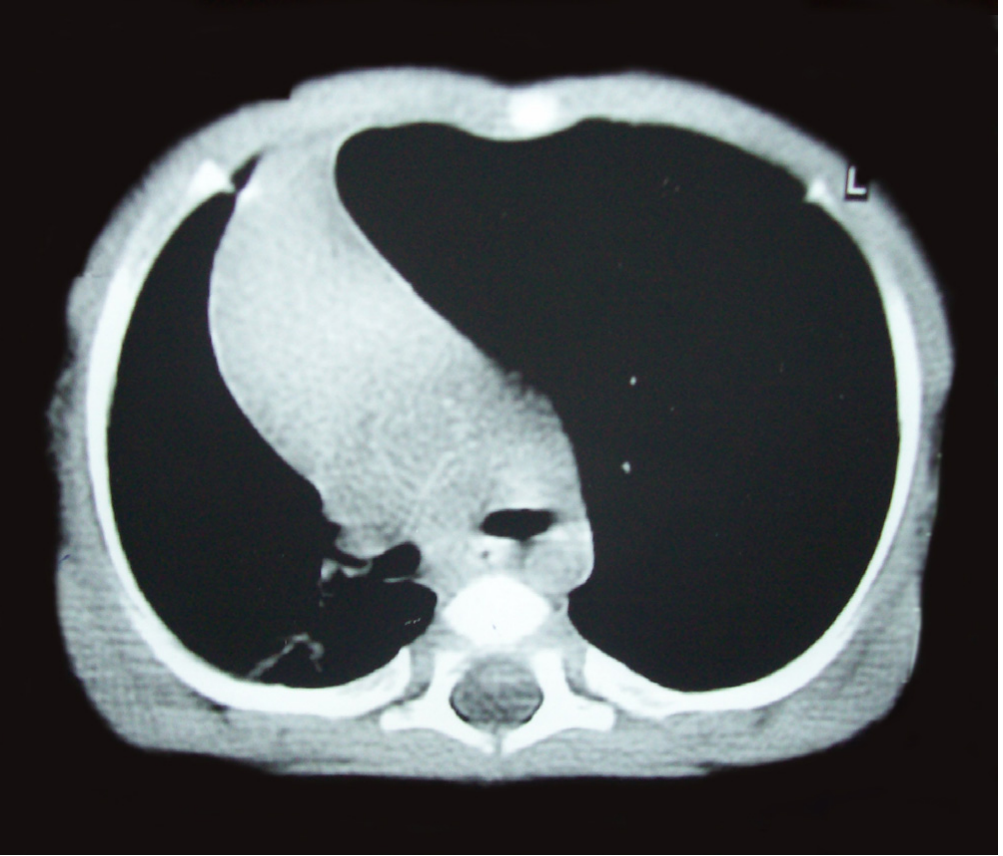
**CT scan showed a hyperluscent, hyper extended lobe with midline substantial herniation and compression of the remaining lung**. The mediastinum is significantly shifted away from the side of the abnormal lobe.

**Figure 4:**
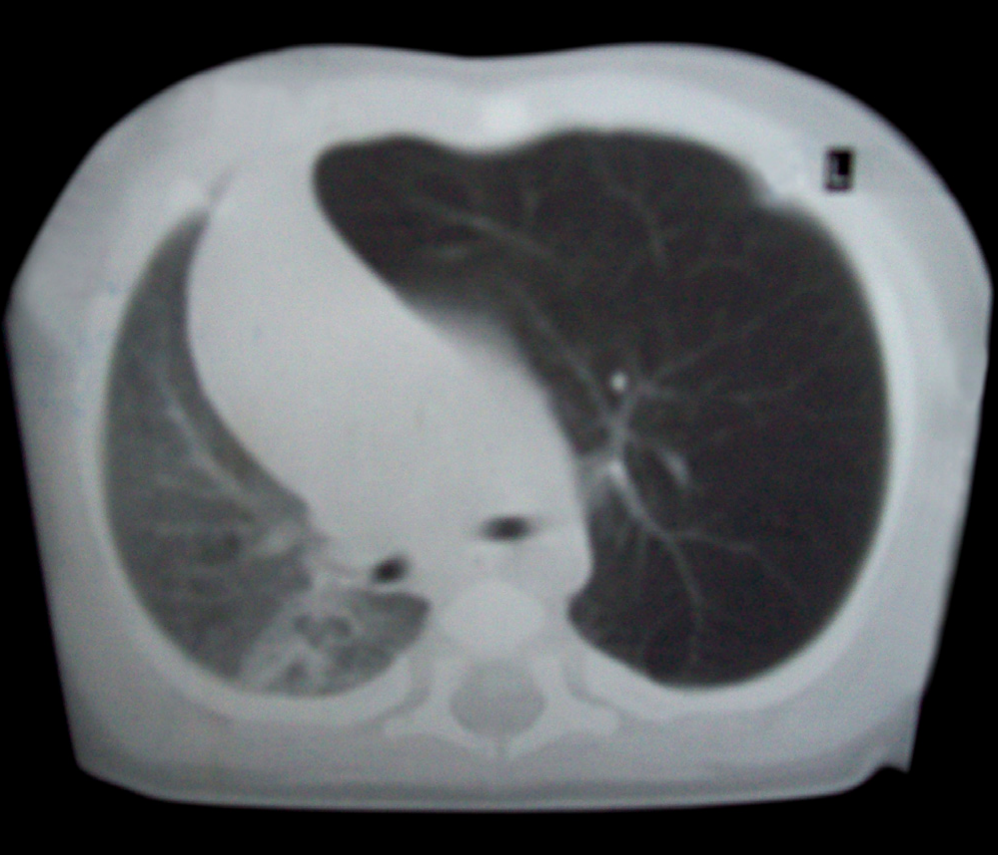
**CT scan showed a hyperluscent, hyper extended lobe attenuated but intact pattern of organized vascularity**.

CT scan showed a hyperluscent, hyper extended lobe (attenuated but intact pattern of organized vascularity) with midline substantial herniation, compression of the remaining lung and the mediastinum is significantly shifted away from the side of the abnormal lobe (Figure 3 and 4).

## Discussion

Our patient had all the features suggestive of the CLE. The baby presented in the neonatal period which is most common. With the investigation facilities available the baby with mild respiratory distress like our case can be diagnosed easily. Antenatal diagnosis can be done with Ultrasonography which was missing in our case [7]. Controversy exists regarding surgical and conservative management of this malformation. There is no contentious opinion. One opinion is in favor of conservative management for mild cases but stringent follow up is necessary [6]. In our case conservative management was given and we are following up the baby. Now the age of the is one and half month. He is normal and growing up as per other baby of same age and sex.

## Conclusion

The early diagnosis of this case and the importance of conservative management rather than advocation of surgery in all CLE cases are noteworthy. The importance of follow up is stressed.

## Competing interests

The authors declare that they have no competing interests.

## Authors' contributions

AKD was responsible for patient care, follow up and drafting of paper; SM collected data and assisted AKD to prepare manuscript, JJ involved in the radiological investigation of the study.

## Consent

Written informed consent was obtained from the patient for publication of this case report and accompanying images. A copy of the written consent is available for review by the Editor–in–Chief of this Journal.
